# SciLite: a platform for displaying text-mined annotations as a means to link research articles with biological data

**DOI:** 10.12688/wellcomeopenres.10210.2

**Published:** 2017-07-10

**Authors:** Aravind Venkatesan, Jee-Hyub Kim, Francesco Talo, Michele Ide-Smith, Julien Gobeill, Jacob Carter, Riza Batista-Navarro, Sophia Ananiadou, Patrick Ruch, Johanna McEntyre

**Affiliations:** 1 Literature Service group, European Molecular Biology Laboratory, European Bioinformatics Institute (EMBL-EBI), Cambridge, UK; 2 SIB Text Mining, Swiss Institute of Bioinformatics, Geneva, Switzerland; 3 National Centre for Text Mining (NaCTeM), Manchester Institute of Biotechnology, Manchester, UK; 4 Bibliomics and Text Mining Group (BiTeM), HES-SO, Geneva, Switzerland

**Keywords:** Open Access, Biocuration, Text-Mining, Data Integration, Semantic Web, Web Annotations, RDF, SPARQL, SciLite

## Abstract

The tremendous growth in biological data has resulted in an increase in the number of research papers being published. This presents a great challenge for scientists in searching and assimilating facts described in those papers. Particularly, biological databases depend on curators to add highly precise and useful information that are usually extracted by reading research articles. Therefore, there is an urgent need to find ways to improve linking literature to the underlying data, thereby minimising the effort in browsing content and identifying key biological concepts.

As part of the development of Europe PMC, we have developed a new platform, SciLite, which integrates text-mined annotations from different sources and overlays those outputs on research articles. The aim is to aid researchers and curators using Europe PMC in finding key concepts more easily and provide links to related resources or tools, bridging the gap between literature and biological data.

## 1. Introduction

Biological databases underpin life sciences research, aiding scientists in the process of knowledge discovery. The data within EMBL-EBI data resources were recently estimated to make research more efficient, minimally by £1bn per year ( Beagrie & Houghton, 2016). One of the major contributing factors to this value is the structured rich metadata around deposited datasets, gathered both on submission and post-deposition, largely through the efforts of professional data curators who extract pertinent information, making the data more useful to the scientific community. Trained expert curators read numerous scientific articles to annotate data with information, such as biological functions, molecular interactions and gene-disease associations. Biocuration follows a formalised workflow that often involves: a) finding domain-relevant articles; b) identifying mentions of the bioentities of interest in those articles, e.g., proteins, genes, diseases, and accession numbers; c) identifying molecular events and evidences, such as entity interactions and experimental methods; and d) coordinating with software developers to update the information and annotations in the databases ( Dauga, 2015; Hirschman *et al.*, 2012). Additionally, curators often collaborate with developers and researchers in developing standards for data collection, nomenclature, vocabularies/ontologies and metadata. Curation is a time consuming and challenging task, as the bio-entities and biological concept descriptions sought be spread within an article or over multiple articles. For instance, curators may have to identify interacting partners for a large set of proteins or protein complexes, categorizing them based on evidences for the interaction, such as physical and/or causal interactions, often inferred through implicit author statements. The International Society for Biocuration (ISB) ( Bateman, 2010) was founded in 2009, to co-ordinate curation activities and share new methods. Considering the exponential growth in data, there is increasing pressure for curation to be better supported by sophisticated computational approaches to make curation efforts sustainable in the long term.

To this end, text-mining methodologies offer one approach to enhance biocuration workflows. Automated information extraction and literature analysis using text-mining has increased in sophistication over the last decade, with the ability to process full text articles, and retrieve entities and relationships, such as accession numbers, molecular interactions and gene-disease associations ( Rebholz-Schuhmann *et al.*, 2012). Dedicated tools and pipelines have been developed for retrieving articles based on a given article category, tagging bio-entities of interest in articles, and identifying co-occurrences of entities in texts based on set of relations ( Ananiadou *et al.*, 2015; Kafkas *et al.*, 2016; Piñero *et al.*, 2015; Pletscher-Frankild *et al.*, 2015). There are a number of text-mining based tools that have been developed to facilitate automated extraction of various article types and biological concepts, such as Textpresso ( Müller *et al.*, 2004), iHOP ( Fernández *et al.*, 2007), Whatizit ( Rebholz-Schuhmann *et al.*, 2008), EAGLi ( Gobeill *et al.*, 2009; Gobeill *et al.*, 2015) EVEX ( Landeghem & Ginter, 2011), PubTator ( Wei *et al.*, 2013) and Argo ( Rak *et al.*, 2014). Among these tools, Textpresso has been significantly adapted by data providers and the curation community to triage articles for curation ( Druzinsky *et al.*, 2016; Van Auken *et al.*, 2012). Collaboration between curators and the text-mining community has been fostered by the BioCreative (Critical Assessment of Information Extraction systems in Biology) workshop series, which broadly focuses on improving text-mining outputs in terms of precision and recall to assist curators. Additionally, infrastructure initiatives, such as BeCalm and OpenMinTed have more recently been established with an aim to orchestrate various text-mining efforts.

In addition to the above examples, other tools annotate entities in scientific articles and link them to the corresponding data sources. For instance, Reflect ( O’Donoghue *et al.*, 2010) and EXTRACT ( Pafilis *et al.*, 2016) are tools that use real-time tagging and augmented browsing approach to tag bio-entities, such as genes, proteins and small molecules described in papers. Whereas, Utopia documents ( Attwood *et al.*, 2010), is a desktop application that links explicit and implicit information on static (PDF) versions of articles to the corresponding online resources. Furthermore, PubAnnotation is an online resource developed by the Data Base Centre for Life Sciences ( DBCLS), which serves as a repository for text annotations. The tool uses text alignment functions to create new annotations or map existing annotations on scientific articles.

We have to acknowledge that articles are read and discovered in different contexts, for example, via locally saved PDFs, literature indexing services and databases, and via publisher’s websites. While the above listed tools can support information extraction and in some cases integration, the annotations generated are hard to share across different platforms. In part this is due to the lack of a common standard to exchange annotations. Although formats like BioC ( Comeau *et al.*, 2013) and Biological Expression Language ( BEL) are used to represent text-mined outputs, they are highly specific to the domain and their uptake is limited beyond the life sciences text mining community. Recently, the World Wide Web Consortium (W3C) proposed the Web Annotation Data Model as a standard to share annotations across different platforms. Based on the Resource Description Framework (RDF), the model can represent text quotes/fragments, links, video segments and images, which are located in a document via a prefix-suffix tagging approach that offers opportunities to mix and show both human and machine-generated annotations on any instance of the same content.

In this article, we describe a new platform, SciLite, developed as part of Europe PMC ( Europe PMC Consortium, 2015) that allows text-mined annotations from any source to be displayed on full text articles. The aim of this platform is to capitalise on the text-mining advances from the research community, bringing the results to a broader audience of readers and developers who might use the results to address additional challenges around information retrieval, visualization and integration. In the context of ELIXIR, the European Infrastructure for life sciences data, of particular interest is the reuse of text mining outputs to make deeper links between the literature and biological data resources, providing more seamless navigation and clear evidence behind curatorial statements.

## 2. Methods

### 2.1. Architecture

SciLite is a platform that allows text-mined annotations from any provider to be highlighted on scientific articles. The platform consolidates various text-mined annotations (see section 2.2) and displays those annotations on full text research articles browsed within Europe PMC webpages. Currently, SciLite operates on full text articles with the license type: CC0, CC-BY, or CC-BY-NC (~ 900000 articles at the time of writing).

Annotations received from contributors are modelled according to the W3C standard Web Annotation Data Model (see section 2.3 for more information). The annotations are stored in a MongoDB database. MongoDB is performant and fetching annotations for a given PMCID is straightforward. Additionally, annotations are stored as RDF in a triple store ( OpenLink Virtuoso version 7.2). RDF is more powerful for graph-based queries; offering an opportunity to explore SciLite annotations in conjunction with other RDF graphs in the Linked Data cloud. The SciLite RDF endpoint is publicly available (see section 5) and contains over 1.4 billion triples (as of May 2017). Details on classes, relations and sample queries are available in the Supplementary material. Furthermore, we plan on providing a public RESTful API within the next few months. 

**Figure 1.**
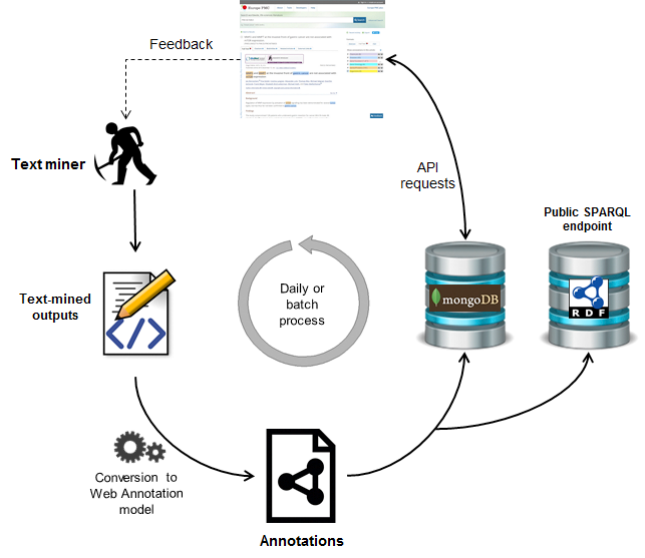
Overview of how text mining results are incorporated into SciLite.

SciLite is very flexible regarding the frequency of data deposition by contributors: anything from a static dataset to daily updates can be accommodated. Once stored in the database, the SciLite application uses an API to retrieve annotations for a given article. The article view provides the opportunity for reader feedback on the quality of annotations, which can be reported back to the source text-mining algorithm for potential future improvement (see section 2.5 for more information). Figure 1 gives an overview of the workflow by which text-mined annotations can be accommodated and viewed on the SciLite platform.

Should it be desirable, the EMBL-EBI Embassy Cloud is available for groups to operate their text-mining workflows on a daily basis.

### 2.2. Annotation types

- **Named entities:** Annotations supplied by the Europe PMC text-mining pipeline identifies concepts, such as gene/protein names, organisms, diseases, GO terms, chemicals and accession numbers. This is achieved through a cascade of three modules: section tagger ( Kafkas *et al.*, 2015), sentence splitter and named entity taggers, a module that applies the dictionary-based approach ( Rebholz-Schuhmann *et al.*, 2008) and a machine-learning based filter ( Chang *et al.*, 2007), for filtering out potential false positives in annotations.
- **Biological events:** The National Centre for Text Mining (NaCTeM), Manchester, UK, extracted annotations on approximately 150,000 open-access articles as part of the Europe PMC project in 2015. For the initial phase, over 400 phosphorylation events from this set are included.
- **Relationships:**Target-disease associations are supplied by two providers:
  - Open Targets ( Koscielny *et al.*, 2017): Open Targets is a platform for accessing potential drug targets associated with disease. The dataset included in SciLite currently contains over 2 million text mined gene-disease associations.
  - DisGeNET ( Piñero *et al.*, 2015): The DisGeNET platform contains collections of genes and gene variants associated with human diseases. The platform contains data from various sources that includes curated sources, animal models and scientific articles. From this, a subset of 7000 gene-disease associations are integrated.
- **Text phrases:**
  - Gene Reference into Function ( GeneRIF): GeneRIF refers to the function of genes that are extracted from articles by MeSH indexers and added to the NCBI’s Entrez Gene records. GeneRIFs can be simple “cut-and-paste” text snippets from abstracts or full text, but sometimes are a more complex synthesis of text fragments. The text mining group at the Swiss Institute of Bioinformatics (SIB), Geneva, Switzerland has mapped GeneRIFs back onto article full text and supplied the results to SciLite as part of the Elixir-EXCELERATE project. A dedicated application has been developed to identify GeneRIFs that combine extracts from several sections of the original article ( Gobeill *et al.*, 2008). The current dataset contains over 32,000 GeneRIF statements.
  - Molecular interactions: Text snippets describing protein-protein interactions extracted manually from scientific articles were supplied by IntAct ( Orchard *et al.,* 2014). Over 100 high-quality curated statements have been included in SciLite.

### 2.3. Representing annotations in SciLite

**Figure 2.**
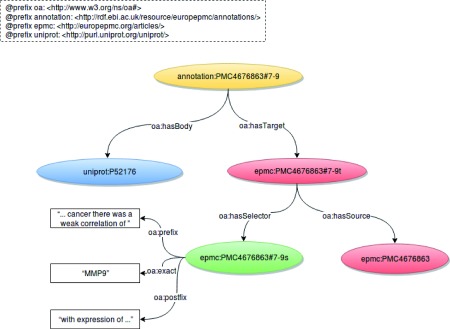
The figure illustrates a sample annotation of protein *MMP9* described in an article ( PMC4676863): the figure lists the vocabularies used to represent the text-mined annotations. The annotation consists of a link for the tagged entity (Body - UniProt: P52176) and the mentions of the entity (Target) in the text snippet. The text is represented by: prefix – the text that occurs before the tagged entity; exact – tagged entity itself ( *MMP9*); and postfix – the text snippet that occurs after the tagged entity.

**Figure 3.**
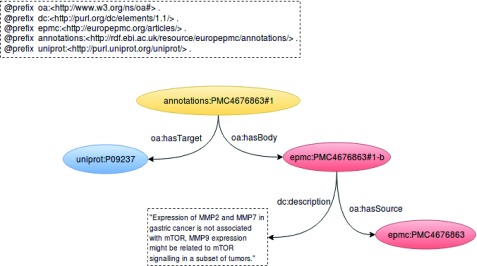
An illustration of a sample GeneRIF (gene function) annotation ( PMC4676863): the figure lists the vocabularies used to represent the annotation. The annotation consists of: Body - text phrase about protein mTOR and a target - data source link for the described protein (UniProt: P09237).

The text-mined outputs are represented in the Web Annotation Data Model. The model consists of a *Target* - the text describing an entity and a *Body* – a link (to the data source) for the tagged entity; the representation may vary according to the type of annotation. For the current set of annotations, we adopted the Text Quote Selector and the Fragment Selector models. The former is used to represent named entities and biological events (see Figure 2) and the latter to represent relationships and text phrases (see Figure 3).

### 2.4. User interface

To make annotations available to readers, the MongoDB database is queried using API requests fetching all the relevant annotations for a given article. The retrieved annotations are highlighted (colour coded) on the Europe PMC website (see Figure 5). Additionally, the annotations are interactive; clicking the highlighted annotation opens a popup-box containing additional information about the annotation (see Figure 6), such as source of annotation and link to the related database. The algorithm used to highlight annotations consists of the following steps:

• Retrieving all the annotations for a specific PMCID from the annotation database using an API request.

• Sorting the annotations from the API response according to their position in the text (ascending order of occurrence). This is done in order to optimize the performance of the searching process in the article text.

• A listener is associated with each annotation tag to display the corresponding information in the popup window.

### 2.5. Improving annotation accuracy

**Figure 4.**
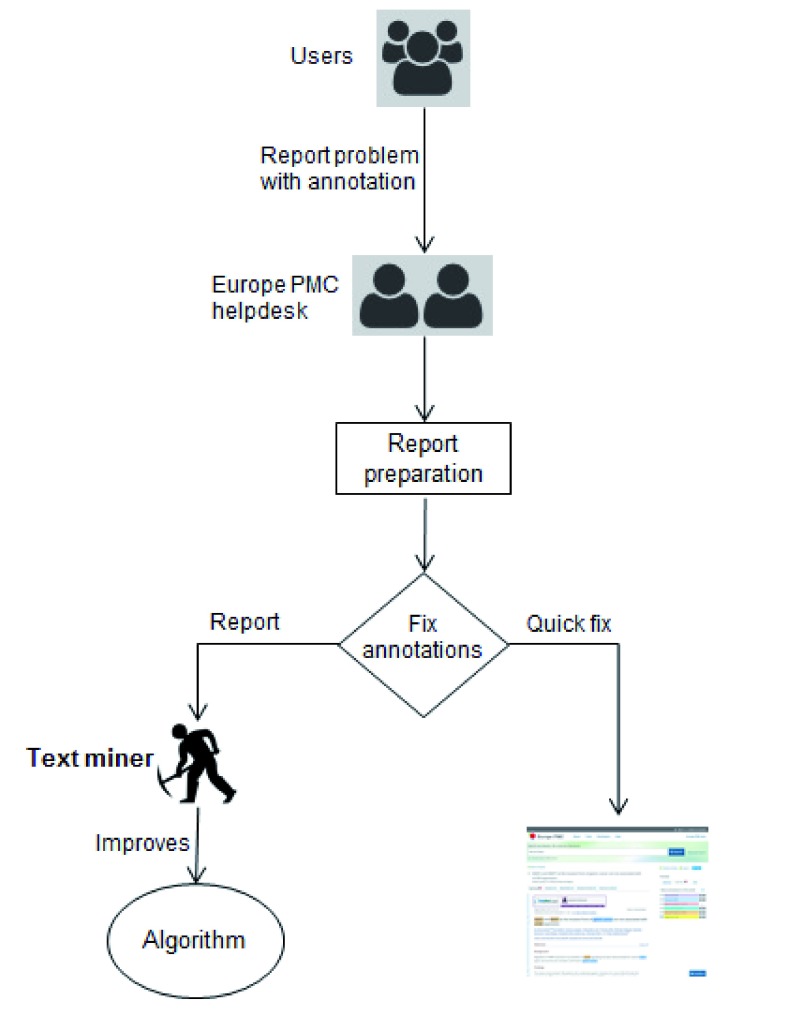
An illustration of the semi-automated feedback mechanism to improve annotations. Erroneous annotations reported by users is used to prepare a report by the helpdesk at Europe PMC. This report is used to perform: a) a quick fix by deleting the particular annotation; b) further the reports are used to refine the text-mining algorithms in the longer term.

To reduce false positives, we have set up a semi-automated process that enables a user to report an annotation error, which triggers the removal of that specific annotation. These reports can be collected periodically to improve the text-mining workflows (see Figure 4). The handling of error reports involves two steps:

- Once an error report is received from a user the particular annotation is deleted from the annotation databases. This is a daily procedure that engenders user trust.
- The report may be used to refine the text-mining algorithms. Often ambiguous terms are reported that are incorrect in the context of the sentence. This often occurs with three letter entity names. For instance, *EPO,* would be tagged as Erythropoietin protein while the sentence could be referring to the *European Patent Office (EPO)*. This step is carried out over a longer period of time, allowing the providers to examine the reports prior to the inclusion of those exceptions in their algorithms.

The current process does not take into account false negatives, i.e. when an annotation was missed by an algorithm. A mechanism to handle false negatives would be a desirable future development.

**Figure 5.**
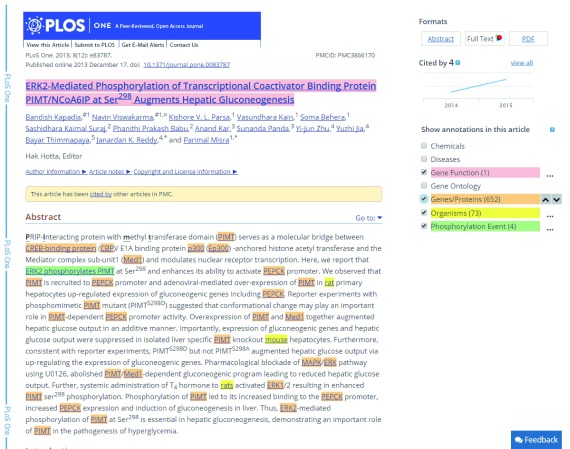
The screenshot shows the front-end rendering of various annotation types for an article on Europe PMC.

## 3. Discussion

 We have adopted a user-driven approach towards the development of SciLite, conducting user research on cognitive and functional aspects of the platform. We have tested SciLite with users that have a range of backgrounds over different geographical locations. A total of 17 users participated, including curators, senior researchers, clinicians and doctoral students. The test sessions were unguided, that is, the users were observed for their behaviour whilst (1) visually scanning an article of interest, (2) discovering annotation types and features and (3) reacting to the annotations. At the end of the session, the subjects were asked to rate the feature:

- Would they like to use such a feature?
- Was the feature easy to use?
- Trust in data quality and confidence in using the feature.

Overall the feedback was positive. While users’ annotation preferences differed based on their background, most people found at least three annotation types (e.g.: gene/proteins, diseases, organisms) useful. Everyone preferred annotations to be turned off by default as the appearance of different colours immediately on viewing the article was distracting. Some users commented that viewing different annotation types highlighted in close proximity in the text was useful, as it suggested a possible relationship between those terms e.g. chemical-disease. In an earlier version of SciLite, users found it hard to locate the highlighted terms in the text. In response to this feedback, we introduced a term navigation feature (up/down buttons) that allows users browse through the highlighted terms quickly. We also found that inaccuracies affects user trust. This observation resulted in the development of the feedback mechanism described in section 2.5. Other useful suggestions addressed the information that appears in the popup window, range of annotation types (e.g.: cell types, software), and the ability to refine a search based on the type of highlighted annotation. The receipt of user feedback and subsequent improvement of SciLite in response is an iterative process that will continue in the future.

### 3.1. Engagement with the text mining community

The goal of the BioCreative v.5 BeCalm task was to provide the means to benchmark text mining services from different providers, addressing not only the efficacy of the text mining itself, but also technical aspects of services offered. Ideally, the outcomes of BeCalm-based benchmarking would feed into SciLite, allowing “best of class” annotations to be made available in Europe PMC interfaces and APIs. Not only would this approach deliver state-of-the-art text mining results into a widely used interface, it would also counter a potential future user interface challenge of too many repeat annotations from different groups layered on articles. While this is technically possible, it could lead to a performance burden and is unlikely to be of interest to the wider scientific community.

Further extended collaborations within the text-mining community will address the challenge of providing different annotation types that serve different user needs. We welcome contributions encourage them to share annotations on SciLite. The SciLite participation page, which provides details on data requirements, submission formats and examples for interested groups.

### 3.2. Future development

**Figure 6.**
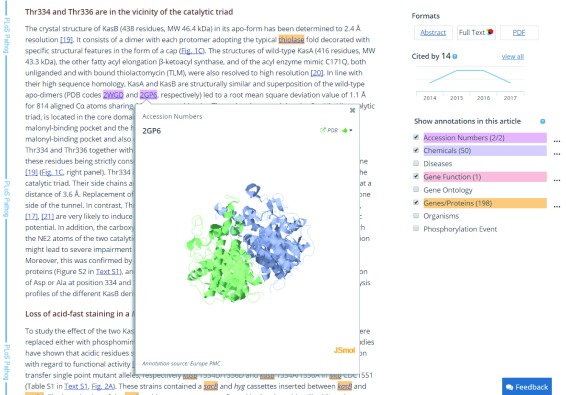
A screenshot showing the 3D molecular structure for a given PDB accession number.

Highlighting biological terms enables skim-reading of articles and the links are useful for verification of the term, but it is only one application of the text mining. We envision that the annotations store and basic SciLite visualisations described here are a basis for the development of further applications by third parties that have the potential to improve full text searching, filtering and integration with biological data. An initial example of this application layer is the use of the BioJS framework to display 3D interactive views of molecular structures identified through text mined PDB accession numbers. In this case, for a given text mined PDB ID, SciLite calls the BioJS module that fetches the structure coordinate information and another that then renders the coordinates using JSmol (see PMC4014462 and Figure 6).

## 4. Conclusion

As there is no let-up in the production of data and publication of articles, the need to find programmatic ways to bridge the gap between literature and data increases. SciLite is an initial step in this direction, but the impact of SciLite will be more pronounced with community-wide participation. We will continue to engage with the text mining and curation communities in particular to extend this work in the future.

## 5. Data and software availability

Annotation examples: Annotations available in SciLite can be accessed here.

Annotation data: The RDF data generated by the SciLite platform is available for querying at: http://www.ebi.ac.uk/europepmc/rdf/sparql

Latest source code: https://github.com/EuropePMC/Biojs.Annotator/tree/Biojs.Annotator_1.0

Archived source code as at the time of publication: Biojs.Annotator version 1.0 - DOI: https://doi.org/10.5281/zenodo.183819 ( Talo' & EuropePMC, 2016).

License: Apache version 2.0

## Supplementary material

**Supplementary File 1: Classes and relations used to model the annotations as RDF, with sample SPARQL queries.**
