# Supplementary material for: SciLite: a platform for displaying text-mined annotations as a means to link research articles with biological data
Source: Wellcome Open Res. 2017 Jul 10;1:25. Originally published 2016 Dec 12. [Version 2] doi: 10.12688/wellcomeopenres.10210.2 (PMC5527546; doi:10.12688/wellcomeopenres.10210.2)
Supplement: Supplementary file 1 [file wellcomeopenres-1-13087-s0000.tgz › 6b1601c6-52b2-4856-a0c1-b127fe7eb5cb.pdf]

Supplementary File 1

# SciLite RDF endpoint:

[http://www.ebi.ac.uk/europepmc/rdf/sparql](http://www.ebi.ac.uk/europepmc/rdf/sparql)

## Named graphs:

| Annotation Source | Graph URI |
|---|---|
| Named entities - Europe PMC | <http://rdf.ebi.ac.uk/dataset/textmining/2016-02-24> |
| Gene function (GeneRIF) - Bibliomics and Text Mining group at the University of Applied Sciences, Geneva (BiTeM) | <http://rdf.ebi.ac.uk/dataset/generif/2016-04-29> |
| Biological events - National Centre for Text Mining (NaCTeM), Manchester, UK | <http://nactem.ac.uk/event_annotations> |

## List of classes:

| Name space | Classes |
|---|---|
| http://www.w3.org/ns/oa# | Annotation |
| | FragmentSelector |
| | SpecificResource |
| | SemanticTag |
| | Tagging |
| | Describing |
| http://purl.org/orb/ | Heading |
| | Introduction |
| | Methods |
| | Results |
| | Discussion |
| http://nactem.ac.uk/schema/uima/typesystem/CancerMechanisms# | uk.ac.nactem.uima.bigm.Event |
| http://rdfs.org/ns/void# | Dataset |

## List of relations:

| Name space | Classes |
|---|---|
| http://www.w3.org/ns/oa# | confirmsTo |
| | exact |
| | postfix |
| | prefix |
| | start |
| | suffix |
| | hasBody |
| | hasRole |
| | hasSelector |

| | |
|---|---|
| | hasTarget |
| | hasSource |
| http://purl.org/orb/ | Header |
| | Introduction |
| | Methods |
| | Results |
| | Discussion |
| http://nactem.ac.uk/schema/uima/typesystem/CancerMechanisms# | uk.ac.nactem.uima.bigm.Concept:id |
| | uk.ac.nactem.uima.bigm.Concept:normalisedName |

## Sample Queries:

The sample queries retrieves annotation for a given PMCID (e.g.: PMC4047089)

### Named entities

Annotation source: Europe PMC

```
PREFIX rdf: <http://www.w3.org/1999/02/22-rdf-syntax-ns#>

PREFIX rdfs: <http://www.w3.org/2000/01/rdf-schema#>

PREFIX oa: <http://www.w3.org/ns/oa#>

PREFIX dcterms: <http://purl.org/dc/terms/>

PREFIX graph: <http://rdf.ebi.ac.uk/dataset/textmining/2016-02-24>

SELECT ?annotation ?position ?tag ?prefix ?exact ?postfix ?section

WHERE {

 GRAPH graph: {

  ?annotation oa:hasBody ?tag.

  ?annotation oa:hasTarget ?target.

  ?target oa:hasSource <http://europepmc.org/articles/[PMCID]> .

  ?target dcterms:isPartOf ?section.

  ?target oa:hasSelector ?selector.

  ?selector oa:exact ?exact.

  ?selector oa:postfix ?postfix.

  ?selector oa:prefix ?prefix.

  BIND (replace(strafter(str(?annotation), "#"), "-", ".") as
?position)

 }
```

```
}
ORDER BY ?position
```

## Gene function (GeneRIF)

Annotation source: Text Mining Group, Swiss Institute of Bioinformatics (SIB), Geneva, Switzerland

```
PREFIX dcterms: <http://purl.org/dc/terms/>
PREFIX dc: <http://purl.org/dc/elements/1.1/>
PREFIX void: <http://rdfs.org/ns/void#>
PREFIX oa: <http://www.w3.org/ns/oa#>
PREFIX graph: <http://rdf.ebi.ac.uk/dataset/generif/2016-04-29>

SELECT ?annotation ?target ?exact ?section
WHERE{
 GRAPH graph: {
   ?annotation oa:hasBody ?body .
   ?annotation oa:hasTarget ?target .
   ?body oa:hasSource <http://europepmc.org/articles/[PMCID]> .
   ?body dcterms:isPartOf ?section .
   ?body dc:description ?exact .
 }
}
```

## Biological events

Annotation source: NaCTeM

```
PREFIX rdfs: <http://www.w3.org/2000/01/rdf-schema#>
PREFIX oa: <http://www.w3.org/ns/oa#>
PREFIX rdf: <http://www.w3.org/1999/02/22-rdf-syntax-ns#>
PREFIX graph: <http://nactem.ac.uk/event_annotations>

SELECT DISTINCT ?annotation ?start ?prefix ?exact ?suffix ?entity_id
?uri ?e_start ?e_prefix ?e_exact ?e_postfix
```

```
WHERE {
 GRAPH graph: {
  ?annotation oa:hasBody ?event_target .
  ?annotation oa:hasTarget ?entity_target.
  ?event_target
<http://nactem.ac.uk/schema/uima/typesystem/CancerMechanisms#uk.ac.nactem.uima.bigm.Concept:normalisedName> ?normName .
  ?event_target oa:hasSource "http://europepmc.org/articles/[PMCID]" .
  ?event_target oa:prefix ?prefix .
  ?event_target oa:exact ?exact .
  ?event_target oa:suffix ?suffix .
  ?event_target oa:start ?start .
  ?entity_target
<http://nactem.ac.uk/schema/uima/typesystem/CancerMechanisms#uk.ac.nactem.uima.bigm.Concept:id> ?entity_id .
  BIND (concat("http://purl.uniprot.org/uniprot/",
substr(?entity_id, 9, 6)) as ?uri)
  ?entity_target oa:exact ?e_exact .
  ?entity_target oa:prefix ?e_prefix .
  ?entity_target oa:suffix ?e_postfix .
  ?entity_target oa:start ?e_start .
 }
}
```
