# Supplementary material for: SciLite: a platform for displaying text-mined annotations as a means to link research articles with biological data
Source: Wellcome Open Res. 2017 Jul 10;1:25. Originally published 2016 Dec 12. [Version 2] doi: 10.12688/wellcomeopenres.10210.2 (PMC5527546; doi:10.12688/wellcomeopenres.10210.2)
Supplement: Peer review history

# Peer review: Referee response for version 2

**DOI:** 10.21956/wellcomeopenres.13087.r24114

**Published:** 2017-07-18

**Authors:** Diana Maynard

**Affiliations:** 1 Department of Computer Science, University of Sheffield, Sheffield, UK

The authors have taken the reviewers' comments into consideration and I am happy with the revised version, other than a few minor English mistakes which should be fixed.

 p.3 on set of relations -> on sets of relations 

 with an aim to orchestrate -> with the aim of orchestrating

 Whereas, Utopia documents -> On the other hand, Utopia documents

 publisher's websites -> publishers' websites

 p.4 "performant" is not an English word. I suggest "perform well" or equivalent.

 p.5 Annotations....identifies concepts -> Annotations..identify concepts

 a subset....are integrated -> a subset...is integrated

 onto article full-text -> onto full-text articles

 EPO, would be tagged -> EPO would be tagged

 p.7 allows users browse -> allows users to browse

 inaccuracies affects -> inaccuracies affect

 p.10 The last 2 sentences in paragraph 2 (beginning "We welcome contributions") are both ungrammatical. Also, encourage whom? Contributors? The second sentence has no main verb.

I have read this submission. I believe that I have an appropriate level of expertise to confirm that it is of an acceptable scientific standard.

# Peer review: Referee response for version 2

**DOI:** 10.21956/wellcomeopenres.13087.r18409

**Published:** 2017-07-18

**Authors:** Lynette Hirschman

**Affiliations:** 1 Biomedical Informatics, MITRE Corporation, Bedford, MA, 01730, USA

This version has addressed the major comments from the reviewers. I have only a few minor suggestions for further revision.

 In Figures 2 and 3, what is the significance of the colors? They don't seem to be consistent across the figures. Also, the presentation order for the children of a given node type seems to differ in the figures -- in Fig 2, the "hasBody" link is the left-hand child and hasTarget is the right-hand child; in Fig 3, those are reversed. Presumably order is not relevant, but it would be easier on the reader if the presentation order were consistent. Also, it would be useful to provide (an excerpt) of the actual text that gave rise to the annotation.

 Section 3: there is reference to a "feature" that subjects were asked to rate. What does "feature" refer to?

 Section 3 5 lines from end of paragraph: I couldn't understand the sentenced "...that appears in the popup window, range of annotation types..."

 p. 10, l. 6 -- "While this is technically possible..." -- what is technically possible? Providing repeat annotations? 

 A few minor editing suggestions:

- The term "evidence" is a collective noun in English; use of the plural sounds awkward.
- p. 3 Introduction col 1, line 4 from bottom: this is hard (for me) to parse - does this mean: ...descriptions sought can be spread within an article..."?
- p. 3 col 2., parag 2, line 10: set should be "sets"
- p. 3 col 2 parag 3 l. 5 _ "an augmented browsing approach"
- p. 4  col 1 line 5: "their uptake is limited outside of the life sciences..."
- p. 4 col 2 parag 2 graph-based queries, offering an ... (change semi-colon to comma)
- p. 5 col 1 line 6 from bottom:  "A dedicated applications has been developed ... that combines extracts (should be singular)
- p. 7 col 2 inaccuracies affect user trust (should be plural form)

.

I have read this submission. I believe that I have an appropriate level of expertise to confirm that it is of an acceptable scientific standard.

# Peer review: Referee response for version 1

**DOI:** 10.21956/wellcomeopenres.10999.r19404

**Published:** 2017-01-17

**Authors:** Diana Maynard

**Affiliations:** 1 Department of Computer Science, University of Sheffield, Sheffield, UK

This paper describes an interesting and useful platform for enhancing research articles with enriched biological metadata, and the ensuing visualisation. It is good to see that both the software and data are also freely available. The description of the architecture and rationale are clearly presented, but I think the paper needs some refocusing. There are many existing similar platforms for displaying such metadata, and thus the concept itself is far from novel. It is unclear how the SciLite approach in principle differs from such existing platforms. The second paragraph of the Introduction provides some references to these, which is used as evidence of the importance of this kind of tool, along with the current development of new infrastructures such as OpenMinTed, but some explanation of how SciLite could (or could not) be integrated with these would be useful.

 What is interesting in this work is the particular focus on the biological data and the underlying approach to annotation, but this is treated rather superficially in the paper.

 Something which concerns me a little is that the approach to annotation itself seems rather shallow – this may simply due to the brevity of description and the focus more on architectural aspects. In the Introduction, Semantic Web Technologies are mentioned, but I recommend going a little bit beyond the concept that “ontologies are useful” and explaining in more detail how they help with the task. From the description of the annotation process and visualisation, this is not apparent, although linking terms to ontologies offers great potential for enhanced visualization and exploration for the end-user (though it’s not clear whether this is offered in the platform, and if not, why not). The authors claim that “when such information is presented effectually it will aid users in identifying the main concepts” but it is not clear how this is possible in the current platform. Note also that “effectually” should be replaced with “effectively”.

 I would suggest expanding Section 2.2 to explain better the methodology for named entity extraction. From the description, the approach sounds rather simplistic, but I suspect that this description hides some complexity. A fuller description of the actual dictionary-based approach and spurious entity filtering techniques would be useful. The reference given is only a summary of dictionary-based approaches in general, but I couldn’t see anywhere a description of or link to the actual technique used in SciLite. It is also not clear how major issues such as entity disambiguation and variation are dealt with, in order to ensure correct linking to the ontology.

 In Figure 2, please make it clearer what the original text is from which the figure is derived.

 Figure 4 does not actually show the popup containing additional information about the annotation, but the text describing it indicates that it does. It would be useful to see this popup also.

 Section 2.5 goes into quite some detail about the interface, but most of this is rather standard technology used in most annotation/text mining GUIs (for instance, the mechanism for sorting the annotations to display) and could easily be omitted. In the last bullet point in this section, “correspondent” should be “corresponding”.

 In Section 2.6 (and throughout the paper), “setup” should be two words when used as a verb. In bullet point b, it is not clear what is meant by “relatively” here – I think this word could just be deleted.

 In Section 3, the presentation of the evaluation could do with being more detailed, as this is an important aspect of the platform. What does it really mean to say that “most people found at least 3 annotation types useful”? This is rather vague. How many annotation types were there in total? Did they find the actual annotations useful too? What about the ontologies and the popup information? How did they use the information shown to them, and how did it enhance their experience? What did they find not useful? Did they have suggestions for improvement? How accurate were the annotations? Are there any plans to provide relational information also (as it seems to be indicated by the users that this would be useful)? There are tools that already can provide this kind of information, see for example GATE’s Prospector tool:

 V. Tablan, K. Bontcheva, I. Roberts, and H. Cunningham. Mímir: An open-source semantic search framework for interactive information seeking and discovery. Journal of Web Semantics: Science, Services and Agents on the World Wide Web, 2014.

 A final point – throughout the paper, the authors have added a comma after the phrase “such as”. The comma should precede this phrase. For example, “web annotations such as, text…” should be “web annotations, such as text…”

 In summary, the work is interesting, but I think it could be enhanced greatly by a considerable rewrite with more focus on the underlying methodology and evaluation parts.

I have read this submission. I believe that I have an appropriate level of expertise to confirm that it is of an acceptable scientific standard, however I have significant reservations, as outlined above.

# Response

**Published:** 2017-07-04

**Authors:** Aravind Venkatesan

**Affiliations:** EMBL European Bioinformatics Institute, UK

Dear Diana Maynard,

Thank you for providing your inputs with regard to our paper. We have reworked the manuscript, addressing your suggestions.

Overview of the changes made are as follows:

- Section 1. Introduction: The section has being restructured and now acknowledges contributions made by other annotation tools.
- Section 2. Methods:
  - Sub-section 2.1 (Architecture) now provides an overview of the architecture of SciLite (includes new Figure 1)
  - Sub-section 2.2 (Annotation types) has been expand to include new annotations types (gene-disease relationships and manually curated protein-protein interactions).
  - Sub-section 2.3 describes the Web Annotation model (includes new Figures: 2 and 3).
  - Sub-section 2.4 (User interface) has been reworked to provide concise description of the highlighting process.
  - Sub-section 2.5 now includes a new Figure (4) that provides an overview on the feedback mechanism. 
- Section 3. Discussion: The section now provides details on how the user research was conducted. The section has been restructured with two new sub sections:
  - 3.1 – Engagement with text-mining community
  - 3.2 – Future directions.
- Supplementary material: The supplementary material provides details on the classes and relations used to model the annotations as RDF along with sample SPARQL queries.

We think that the manuscript is much improved as a result of your feedback. Please see a point-by-point response, below:

*The description of the architecture and rationale are clearly presented, but I think the paper needs some refocusing. There are many existing similar platforms for displaying such metadata, and thus the concept itself is far from novel. It is unclear how the SciLite approach in principle differs from such existing platforms. The second paragraph of the Introduction provides some references to these, which is used as evidence of the importance of this kind of tool, along with the current development of new infrastructures such as OpenMinTed, but some explanation of how SciLite could (or could not) be integrated with these would be useful.

The Introduction section now acknowledges the state of the art and describes the complementary role of SciLite.

The novelty of SciLite is that it allows annotations from multiple sources to be included and displayed in Europe PMC, for the purposes of reuse for readers, and to integrate literature with data on an infrastructural level. Europe PMC is a database that is updated daily with new content and already has a large user base, so rather than expecting users to visit another interface, the text-mining results are incorporated into their usual search behaviour. SciLite complements OpenMinTed in that the outputs from such infrastructures, which is focussed on the needs of text miners rather than end users, can be made widely and publically available. This aspect has been included in section 3.1.

*What is interesting in this work is the particular focus on the biological data and the underlying approach to annotation, but this is treated rather superficially in the paper.

*Something which concerns me a little is that the approach to annotation itself seems rather shallow – this may simply due to the brevity of description and the focus more on architectural aspects. In the Introduction, Semantic Web Technologies are mentioned, but I recommend going a little bit beyond the concept that “ontologies are useful” and explaining in more detail how they help with the task. From the description of the annotation process and visualisation, this is not apparent, although linking terms to ontologies offers great potential for enhanced visualization and exploration for the end-user (though it’s not clear whether this is offered in the platform, and if not, why not). The authors claim that “when such information is presented effectually it will aid users in identifying the main concepts” but it is not clear how this is possible in the current platform. Note also that “effectually” should be replaced with “effectively”.*

We acknowledge that some of the original text was misleading. In the current version of the manuscript parts of the introduction has been reworked, focussing on sharing of text-mined annotations for the benefit of the end-user. In this regard, we have now added a paragraph to review the state of the art, highlighting the advantages of using the Web Annotation Data model and the complementary role played by SciLite.

*I would suggest expanding Section 2.2 to explain better the methodology for named entity extraction. From the description, the approach sounds rather simplistic, but I suspect that this description hides some complexity. A fuller description of the actual dictionary-based approach and spurious entity filtering techniques would be useful. The reference given is only a summary of dictionary-based approaches in general, but I couldn’t see anywhere a description of or link to the actual technique used in SciLite. It is also not clear how major issues such as entity disambiguation and variation are dealt with, in order to ensure correct linking to the ontology.

The main aim of SciLite is to be a platform for sharing the text mining outputs multiple sources, in this sense, describing text mining methodology used by contributors is beyond the objectives of SciLite. Since the Europe PMC text mining pipeline also serves as a provider for SciLite we briefly mention the steps involved, citing the articles in which the methodology of these steps are described.

*In Figure 2, please make it clearer what the original text is from which the figure is derived.

We have simplified Figures 2 and 3. The figure legends now describe the sample model with links to the source articles that contains the original annotation.

*Figure 4 does not actually show the popup containing additional information about the annotation, but the text describing it indicates that it does. It would be useful to see this popup also.

The correction has now been made, the text now refers to Figure 6 which includes a popup window and an example link has been included for the same.

*Section 2.5 goes into quite some detail about the interface, but most of this is rather standard technology used in most annotation/text mining GUIs (for instance, the mechanism for sorting the annotations to display) and could easily be omitted. In the last bullet point in this section, “correspondent” should be “corresponding”.

We agree with the point being made. We have now reworked the section providing concise description of the highlighting process.

*In Section 2.6 (and throughout the paper), “setup” should be two words when used as a verb.

We have now made the necessary corrections throughout the article.

*In bullet point b, it is not clear what is meant by “relatively” here – I think this word could just be deleted.

Step b requires the providers to make necessary improvements to their algorithms and this is more time consuming than step a, which is a “quick fix”. The text has been rephrase, we hope the sentence reads better.

*In Section 3, the presentation of the evaluation could do with being more detailed, as this is an important aspect of the platform. What does it really mean to say that “most people found at least 3 annotation types useful”? This is rather vague. How many annotation types were there in total? Did they find the actual annotations useful too? What about the ontologies and the popup information? How did they use the information shown to them, and how did it enhance their experience? What did they find not useful? Did they have suggestions for improvement? How accurate were the annotations?

We agree with the point being made with regards to user research. Section 3 has been expanded, providing details on how the usability tests were conducted, aspects of SciLite that the users liked and how we improved SciLite based on the feedback we received.

*Are there any plans to provide relational information also (as it seems to be indicated by the users that this would be useful)?

SciLite now includes gene-disease relationship provided by two contributors (Open Targets platform and DisGeNET) and protein-protein interactions from IntAct. To accommodate the latest development we have expanded sub-section 2.2

*There are tools that already can provide this kind of information, see for example GATE’s Prospector tool:

*V. Tablan, K. Bontcheva, I. Roberts, and H. Cunningham. Mímir: An open-source semantic search framework for interactive information seeking and discovery. Journal of Web Semantics: Science, Services and Agents on the World Wide Web, 2014.*

We have now reworked the manuscript focusing on SciLite as a platform for bringing various text mined annotations to the wider scientific community. In this sense, we believe that Mímir (as a search framework) is broader in its application when compared to SciLite.

*A final point – throughout the paper, the authors have added a comma after the phrase “such as”. The comma should precede this phrase. For example, “web annotations such as, text…” should be “web annotations, such as text…”

We have edited the text for grammatical corrections and hope that the text now reads significantly better!

# Peer review: Referee response for version 1

**DOI:** 10.21956/wellcomeopenres.10999.r18964

**Published:** 2017-01-13

**Authors:** Lee Harland

**Affiliations:** 1 Scibite Limited, Cambridge, UK

* This article describes the "SciLite" tool for enriching biomedical articles with annotations derived from text mining and other sources and the benefits such an approach has for users. It is well written and referenced and outlines the principles in a consistent way. The application links at the bottom of the article pointed to valid working websites. I think it valid to review both the paper and the tool itself and all of the functionality I tried on the website worked very well and there were no software errors, validating that the system is running as described in the paper.

 In terms of areas for consideration:

 * The introduction is fine, the major omission being coverage of previous work on in-line biological annotation, I would strongly support the acknowledgement of the reflect.ws tool which was perhaps the most prominent one here - and note that many biological web page annotators, both commercial and non-commercial exist (e.g. EXTRACT https://academic.oup.com/database/article/doi/10.1093/database/baw005/2630147/EXTRACT-interactive-extraction-of-environment which works very well on the Europe PMC website). The introduction should clarify the advantages the authors approach brings over 3rd party, javascript bookmarklet approaches.

 * Its not clear what the actual tagging methodology is 2.2 states "Named entity taggers: the module is based on the dictionary-based approach (Rebholz-Schuhmann *et al.*, 2008) combined with a machine-learning based filter (Chang *et al.*, 2007), for filtering out potential false positives in annotations.". Does this use these existing tools or have the authors invented new ones and which vocabularies were used? I think those in the text mining field would be interested to know more about how the text mining is done [I would agree this is not a text mining methods paper, but this is very light on detail here]. Maybe I missed it but "Sentence splitter: an in-house module to identify sentence boundaries." could also do with some form of definition (what's it based on, were any improvements needed etc, the tool correctly doesn't split 'Nkx6.1' so there is some logic being coded there).

 * The use of RDF is interesting but no justification is given as to what advantages RDF provides over other database mechanisms. Why was RDF chosen over other systems? It seems that the queries are fairly straight forward (for an input ID give me annotations) and I'm not sure they require RDF necessarily. There are genuine other reasons I could think of but it would be good for the authors to describe this choice. Indeed the authors then state they are considering use of MongoDb (using RDF or not?). This seems to hint at some problems using RDF, but no information is given. I think the authors either need to describe why or perhaps remove this sentence entirely as these seems like more internal technical discussion?

 * The system performed well for human centric articles but less so when looking at plant or bacterial data... This is a really hard problem in text mining and I don't expect the authors to solve it here but it would be good to perhaps outline whether the system could be tuned to address anything here as non-human area researchers will be using the tool.

 * 2.6.a When error reported there is a "Quick fix" where the annotation is deleted. It is not clear if this is just for that 1 article or all articles as it may be an erroneous synonym? While 2.6.b hints to this "refine the  text-mining algorithm." it gives no detail as to what this entails. Again, this is not a text mining methods paper, but examples would be useful here.

 * Further, Figure 1 - doesnt touch on maintainence. When source vocabs change (e.g. as 2.6.b hints) do all articles need to be re-run? Are there version markers on the annotations? How regularly are older articles re-annotated?  Is provenance applied to annotations, I didn't see any void/prov markers in Figure 2.

 * Following on the provenance theme, the authors state: "Additionally, the open architecture of SciLite... data for clear provenance of curatorial statements.". I think this is shown in Figure 3 but only if you know what the RDF prefixes for void and prov mean, so perhaps should be described in the main article text for non RDF people.

 * Finally, section (2) talks about the systems handling of false positives but does not mention false negatives at all which are equally irritating to the user. What is the proposed approach here?

 * 3. "Everyone preferred annotations to be turned off by default." - I think thats a fairly important statement, did you ask why?

 * The link to the Sparql end point http://www.ebi.ac.uk/europepmc/rdf/sparql provides only a default view and it is left to the user to figure out the schema and queries necessary. I am not sure of the best mechanism (supplementary data or footnote?) but an example query or two would be very helpful to get started

 * In general, figures could do with a bit more description as to what they are showing.

I have read this submission. I believe that I have an appropriate level of expertise to confirm that it is of an acceptable scientific standard, however I have significant reservations, as outlined above.

# Response

**Published:** 2017-07-04

**Authors:** Aravind Venkatesan

**Affiliations:** EMBL European Bioinformatics Institute, UK

Dear Lee Harland,

Thank you for providing your inputs with regard to our paper. We have reworked the manuscript, addressing your suggestions.

Overview of the changes made are as follows:

- Section 1. Introduction: The section has being restructured and now acknowledges contributions made by other annotation tools.
- Section 2. Methods:
  - Sub-section 2.1 (Architecture) now provides an overview of the architecture of SciLite (includes new figure 1)
  - Sub-section 2.2 (Annotation types) has been expand to include new annotations types (gene-disease relationships and manually curated protein-protein interactions).
  - Sub-section 2.3 describes the Web Annotation model (includes new figures: 2 and 3).
  - Sub-section 2.4 (User interface) has been reworked to provide concise description of the highlighting process.
  - Sub-section 2.5 now includes a new figure (4) that provides an overview on the feedback mechanism. 
- Section 3. Discussion: The section now provides details on how the user research was conducted. The section has been restructured with two new sub sections:
  - 3.1 – Engagement with text-mining community
  - 3.2 – Future directions.
- Supplementary material: The supplementary material provides details on the classes and relations used to model the annotations as RDF along with sample SPARQL queries.

We think that the manuscript is much improved as a result of your feedback. Please see a point-by-point response, below:  

* The introduction is fine, the major omission being coverage of previous work on in-line biological annotation, I would strongly support the acknowledgement of the reflect.ws tool which was perhaps the most prominent one here - and note that many biological web page annotators, both commercial and non-commercial exist (e.g. EXTRACT https://academic.oup.com/database/article/doi/10.1093/database/baw005/2630147/EXTRACT-interactive-extraction-of-environment which works very well on the Europe PMC website). The introduction should clarify the advantages the authors approach brings over 3rd party, javascript bookmarklet approaches.

We have now added a paragraph in the Introduction section acknowledging the contributions made by other annotation tools. The section highlights the rationale behind the development of SciLite in comparison with other tools.

The main advantage of SciLite is that the platform aggregates annotations made by different tools. For instance, if desired annotations made by Reflect or Extract can be shared in SciLite. This allows users to view different types of annotations for a given article on Europe PMC website.

* Its not clear what the actual tagging methodology is 2.2 states "Named entity taggers: the module is based on the dictionary-based approach (Rebholz-Schuhmann et al., 2008) combined with a machine-learning based filter (Chang et al., 2007), for filtering out potential false positives in annotations.". Does this use these existing tools or have the authors invented new ones and which vocabularies were used? I think those in the text mining field would be interested to know more about how the text mining is done [I would agree this is not a text mining methods paper, but this is very light on detail here]. Maybe I missed it but "Sentence splitter: an in-house module to identify sentence boundaries." could also do with some form of definition (what's it based on, were any improvements needed etc, the tool correctly doesn't split 'Nkx6.1' so there is some logic being coded there).

The purpose of SciLite is to be a platform for sharing the text mining work of others, so in a sense the text mining methodology used by contributors is not the business of SciLite. However, as the Europe PMC text mining pipeline also serves as a provider for SciLite we have briefly mentioned the steps involved and referred to the articles in which the methodology of these steps is described.

* The use of RDF is interesting but no justification is given as to what advantages RDF provides over other database mechanisms. Why was RDF chosen over other systems? It seems that the queries are fairly straight forward (for an input ID give me annotations) and I'm not sure they require RDF necessarily. There are genuine other reasons I could think of but it would be good for the authors to describe this choice. Indeed the authors then state they are considering use of MongoDb (using RDF or not?). This seems to hint at some problems using RDF, but no information is given. I think the authors either need to describe why or perhaps remove this sentence entirely as these seems like more internal technical discussion?

We agree with the point being made. Based on our performance tests we find MongoDB to be more performant for retrieving annotations for a given PMCID. Whereas, RDF is a natural choice for linked data and is suitable for performing graph-based queries.   

We have now restructured parts of the section to highlight this point, correspondingly we have changed Figure 1.   

* The system performed well for human centric articles but less so when looking at plant or bacterial data... This is a really hard problem in text mining and I don't expect the authors to solve it here but it would be good to perhaps outline whether the system could be tuned to address anything here as non-human area researchers will be using the tool.

Our approach to resolving the challenges of text mining across different fields and phylogenies is to engage the text mining community to provide their insights and solutions in SciLite. This key message of the article has been reworked to be much clearer (we acknowledge that some of the original phrasing was misleading).

* 2.6.a When error reported there is a "Quick fix" where the annotation is deleted. It is not clear if this is just for that 1 article or all articles as it may be an erroneous synonym?

“Quick fix” is only for the specific instance in a specific article where an error was reported, as means of a quick response to user feedback. We have another mechanism which allows providers to correct their algorithms to reduce errors globally. This method could be improved further in the future to extend the deletion of a specific instance to “all instances in the article”, for example.

*While 2.6.b hints to this "refine the text-mining algorithm." it gives no detail as to what this entails. Again, this is not a text mining methods paper, but examples would be useful here.

Error reports are submitted to the corresponding provider as refining text mining algorithms is not part of the SciLite workflow. However, improvements would entail, for instance, revising dictionaries by removing erroneous synonyms.

* Further, Figure 1 - doesnt touch on maintenance. When source vocabs change (e.g. as 2.6.b hints) do all articles need to be re-run? Are there version markers on the annotations? How regularly are older articles re-annotated?  Is provenance applied to annotations, I didn't see any void/prov markers in Figure 2.

In SciLite when new dataset is submitted, all annotations are checked and in cases where the annotations have changed the corresponding collections (in MongoDB) and the RDF triples are updated. When new data set is available the provenance graphs are updated. 

Figure 1 has been replaced with a new Figure, the figure now provides an overview of the SciLite workflow, touching upon the maintenance aspect.

Figure 2 has been modified to refocus on the representation of annotations in the Web Annotation model. We hope the new figure is more understandable to the readers.

* Following on the provenance theme, the authors state: "Additionally, the open architecture of SciLite... data for clear provenance of curatorial statements.". I think this is shown in Figure 3 but only if you know what the RDF prefixes for void and prov mean, so perhaps should be described in the main article text for non RDF people.

In the indicated sentence we were referring to annotations as textual evidence and were not referring to “provenance” in the context of RDF. We acknowledge that the usage of the phrase was misleading, we have now rephrased the sentence.

* Finally, section (2) talks about the systems handling of false positives but does not mention false negatives at all which are equally irritating to the user. What is the proposed approach here?

You have hit on a tricky problem: how to report a false negative. Without an easy way for people to, for example, highlight text and add a comment, it will be a cumbersome task to report false negatives. It is possible that solutions such as hypothes.is could help here, especially given that both SciLite and  hypothes.is  use the Web Annotation standard; we plan to explore these options in the future.

* 3. "Everyone preferred annotations to be turned off by default." - I think thats a fairly important statement, did you ask why?

Based on the user feedback, we find that annotations highlighted (in different colours) by default are distracting to the user. Hence, the users preferred to choose the type of annotations that are of interest to them.

We have expanded the section explaining the process of testing SciLite with the users and describe the feedback we received.

* The link to the Sparql end point http://www.ebi.ac.uk/europepmc/rdf/sparql provides only a default view and it is left to the user to figure out the schema and queries necessary. I am not sure of the best mechanism (supplementary data or footnote?) but an example query or two would be very helpful to get started

We agree with the point being made, we have now added Supplementary material with details of relations and classes used along with sample queries. We also plan on releasing a RESTful API on the annotations within the next few months

* In general, figures could do with a bit more description as to what they are showing.

We have extended the figure legends to be more descriptive.

# Peer review: Referee response for version 1

**DOI:** 10.21956/wellcomeopenres.10999.r18949

**Published:** 2017-01-05

**Authors:** Lynette Hirschman

**Affiliations:** 1 Biomedical Informatics, MITRE Corporation, Bedford, MA, 01730, USA

The article describes the SciLite project and capability; SciLite opens exciting new possibilities by supporting the automated tagging (and indexing) of biological entities. The article presents the background behind the development of these capabilities and a description of both the capabilities and the underlying components.

 Overall, the article provides a clear presentation of the rationale for SciLite, a useful description of the underlying approach and architecture, and a good bibliography referencing many of the key works related to automated extraction of bio-entities. However, there are several places which need clarification or elaboration, including 1) whether SciLite can extract the “essence” of an article; 2) explanations of the contents (and abbreviations) of Figs 2 and 3 – and how they relate to the text descriptions in the article; 3) some discussion of how to handle multiple sources for the same information, and 4) a link to (a demo of) SciLite, to allow interested people to explore this new capability. In addition, there are some detailed minor questions/suggestions at the end of the review.

  

1. Capturing the “essence” of an article: In the description of capabilities, there is one statement that requires either rethinking or further explanation: “When such information [referring to text-mined, linked entities] is presented effectually it will aid users in identifying the main concepts, plausibly beginning to reduce the burden of extracting the essence of a given article.” (p. 2, col. 2, para. 3) There is a danger that tagging of entities in full text articles can overwhelm the user, rather than giving them a sense of the essence of an article. It may be true that tagging entities in an **abstract** might make the key entities jump out; or that providing a summary table listing the most frequently mentioned entities might provide such an overview of “main concepts” – but simply tagging many types of entities (especially if there are false positives in the tagging) can quickly overwhelm the user.
2. Explaining Figs 2 and 3: These figures each need a paragraph or so of text to explain what is being illustrated, and how to read the graph displayed. For Fig 2., it would be very helpful to see the actual annotation generated (in its textual context); it would be good to gloss the various abbreviations in the graph (what is oa as a prefix? What is orb? What are the urls and their suffixes #1-2s and #1-2t? What are the things in boxes? In the box on the lower left, Fig 2, why are there multiple quote marks: “CSF-1””induces expression…” and what do these mean? 
  For Fig 3, what are the items in the box with dashed lines in the upper left? What do the various terms dc, dcterms, orb, oa, void, prov mean? What is the actual example GeneRIF that is being constructed? And pretty much the same request as for Fig 2 – describe the contents of the figure in text, so that the reader can follow what is being shown. 
3. Handling of multiple sources of the same kind of information – given that there are multiple taggers for certain entities types (e.g., genes/proteins), will there be a capability for users to choose which tagger they want? Will there be a way for users to select high precision vs high recall? Will there be any attempt to create an ensemble system from multiple taggers?
4. Please provide a link so that interested readers can explore SciLite. 

 

 Minor comments/questions:

 p.2 col. 1, para 1 middle – I couldn’t follow the second part of the sentence, starting with “where…”: “This workflow is highly interconnected and precise where the databases are both dependent and required for this practice.”

 p.2 col. 2 para 1: suggest replacing “instituted” with “fostered”.

 p.2. col. 2 para 2 top- suggest removing the first clause (it’s redundant) – e.g., remove “Having described the importance of information extraction by using text-mining methods in the context of aiding manual curation” – and just start the paragraph with “The other essential aspect…”

 p.2, col. 2 para 2 end: suggest changing “and integrate data” to “and integration of data”

 p. 2, col. 2, para 3 l 2: suggest changing “mechanism toward” to “mechanism to support”

 p.2. col. 2 last para: suggest changing “an aim to leverage text-mining solutions towards” to “an aim of leveraging text-mining solutions for”

 p. 5, section 2.5 on User Interface – would it be appropriate to cite the Reflect project here, as a groundbreaking application to provide enriched metadata about objects in text? [ Reflect: augmented browsing for the life scientist E Pafilis, SI O'Donoghue, LJ Jensen, H Horn, M Kuhn… - Nature biotechnology, 2009)

 p. 7, col. 2, para 2 suggest changing “platform, this offers” to “platform, which offers”

 p. 9, col 1 Author Contributions – insert “the” before SciLite text mining pipeline”

I have read this submission. I believe that I have an appropriate level of expertise to confirm that it is of an acceptable scientific standard, however I have significant reservations, as outlined above.

# Response

**Published:** 2017-07-04

**Authors:** Aravind Venkatesan

**Affiliations:** EMBL European Bioinformatics Institute, UK

Dear Lynette Hirschman,

Thank you for providing your inputs with regard to our paper. We have reworked the manuscript, addressing your suggestions.

Overview of the changes made are as follows:

- Section 1. Introduction: The section has being restructured and now acknowledges contributions made by other annotation tools.
- Section 2. Methods:
  - Sub-section 2.1 (Architecture) now provides an overview of the architecture of SciLite (includes new figure 1)
  - Sub-section 2.2 (Annotation types) has been expand to include new annotations types (gene-disease relationships and manually curated protein-protein interactions).
  - Sub-section 2.3  describes the Web Annotation model (includes new figures: 2 and 3).
  - Sub-section 2.4 (User interface) has been reworked to provide concise description of the highlighting process.
  - Sub-section 2.5 now includes a new figure (4) that provides an overview on the feedback mechanism. 
- Section 3. Discussion: The section now provides details on how the user research was conducted. The section has been restructured with two new sub sections:
  - 3.1 – Engagement with text-mining community
  - 3.2 – Future directions.
- Supplementary material: The supplementary material provides details on the classes and relations used to model the annotations as RDF along with sample SPARQL queries.

We think that the manuscript is much improved as a result of your feedback. Please see a point-by-point response, below:

*Capturing the “essence” of an article: In the description of capabilities, there is one statement that requires either rethinking or further explanation: “When such information [referring to text-mined, linked entities] is presented effectually it will aid users in identifying the main concepts, plausibly beginning to reduce the burden of extracting the essence of a given article.” (p. 2, col. 2, para. 3) There is a danger that tagging of entities in full text articles can overwhelm the user, rather than giving them a sense of the essence of an article. It may be true that tagging entities in an abstract might make the key entities jump out; or that providing a summary table listing the most frequently mentioned entities might provide such an overview of “main concepts” – but simply tagging many types of entities (especially if there are false positives in the tagging) can quickly overwhelm the user.

We understand and agree with the point being made. Highlighting different annotation types could serve as an indicator (referred to in the text as “essence”) with respect to the key concepts described in a given paper. We have now rewritten the paragraph to avoid confusion.

*Explaining Figs 2 and 3: These figures each need a paragraph or so of text to explain what is being illustrated, and how to read the graph displayed. For Fig 2., it would be very helpful to see the actual annotation generated (in its textual context); it would be good to gloss the various abbreviations in the graph (what is oa as a prefix? What is orb? What are the urls and their suffixes #1-2s and #1-2t? What are the things in boxes? In the box on the lower left, Fig 2, why are there multiple quote marks: “CSF-1””induces expression…” and what do these mean?

*For Fig 3, what are the items in the box with dashed lines in the upper left? What do the various terms dc, dcterms, orb, oa, void, prov mean? What is the actual example GeneRIF that is being constructed? And pretty much the same request as for Fig 2 – describe the contents of the figure in text, so that the reader can follow what is being shown.*

We agree that the figures could be confusing to readers with limited exposure to RDF concepts. We now briefly describe the Web Annotation Model (Sub-section 2.3), further, we have simplified Figures 2 and 3. The figure legends now describes the sample model with links that point to the source articles that contains the exemplified annotations. 

*Handling of multiple sources of the same kind of information – given that there are multiple taggers for certain entities types (e.g., genes/proteins), will there be a capability for users to choose which tagger they want? Will there be a way for users to select high precision vs high recall? Will there be any attempt to create an ensemble system from multiple taggers?

SciLite can host similar semantic types from multiple sources. The platform allows users to choose relevant sources of their interest. However, it is unlikely that this would be of interest to the wider scientific community. Ideally SciLite would consume annotations through systems like BeCalm, allowing users access to benchmarked “high quality” annotations.   

*Please provide a link so that interested readers can explore SciLite.

SciLite platform is integrated in Europe PMC and can be seen in action on any CC-BY or CC-BY-NC article. We have provided links to the illustrated examples in the figure legends of 2, 3 and 5 and a link (in sub-section 2.1 and section 5) that lists all the CC-BY and CC-BY-NC articles.

*Minor comments/questions:

*p.2 col. 1, para 1 middle – I couldn’t follow the second part of the sentence, starting with “where…”: “This workflow is highly interconnected and precise where the databases are both dependent and required for this practice.”*

*p.2 col. 2 para 1: suggest replacing “instituted” with “fostered”.*

*p.2. col. 2 para 2 top- suggest removing the first clause (it’s redundant) – e.g., remove “Having described the importance of information extraction by using text-mining methods in the context of aiding manual curation” – and just start the paragraph with “The other essential aspect…”*

*p.2, col. 2 para 2 end: suggest changing “and integrate data” to “and integration of data”*

*p. 2, col. 2, para 3 l 2: suggest changing “mechanism toward” to “mechanism to support”*

*p.2. col. 2 last para: suggest changing “an aim to leverage text-mining solutions towards” to “an aim of leveraging text-mining solutions for”*

*p. 5, section 2.5 on User Interface – would it be appropriate to cite the Reflect project here, as a groundbreaking application to provide enriched metadata about objects in text? [Reflect: augmented browsing for the life scientist E Pafilis, SI O'Donoghue, LJ Jensen, H Horn, M Kuhn… - Nature biotechnology, 2009)*

*p. 7, col. 2, para 2 suggest changing “platform, this offers” to “platform, which offers”*

*p. 9, col 1 Author Contributions – insert “the” before SciLite text mining pipeline”*

We have made all the minor correction and added paragraphs to review the state of the art (including the Reflect application).
